# B cell receptor signaling and associated pathways in the pathogenesis of chronic lymphocytic leukemia

**DOI:** 10.3389/fonc.2024.1339620

**Published:** 2024-02-26

**Authors:** Vera K. Schmid, Elias Hobeika

**Affiliations:** Institute of Immunology, Ulm University, Ulm, Germany

**Keywords:** chronic lymphocytic leukemia (CLL), B cell receptor (BCR) signaling, IGHV, CD79a/b (Igα/Igβ), PI3K/AKT, SYK, BTK

## Abstract

B cell antigen receptor (BCR) signaling is a key driver of growth and survival in both normal and malignant B cells. Several lines of evidence support an important pathogenic role of the BCR in chronic lymphocytic leukemia (CLL). The significant improvement of CLL patients’ survival with the use of various BCR pathway targeting inhibitors, supports a crucial involvement of BCR signaling in the pathogenesis of CLL. Although the treatment landscape of CLL has significantly evolved in recent years, no agent has clearly demonstrated efficacy in patients with treatment-refractory CLL in the long run. To identify new drug targets and mechanisms of drug action in neoplastic B cells, a detailed understanding of the molecular mechanisms of leukemic transformation as well as CLL cell survival is required. In the last decades, studies of genetically modified CLL mouse models in line with CLL patient studies provided a variety of exciting data about BCR and BCR-associated kinases in their role in CLL pathogenesis as well as disease progression. BCR surface expression was identified as a particularly important factor regulating CLL cell survival. Also, BCR-associated kinases were shown to provide a crosstalk of the CLL cells with their tumor microenvironment, which highlights the significance of the cells’ milieu in the assessment of disease progression and treatment. In this review, we summarize the major findings of recent CLL mouse as well as patient studies in regard to the BCR signalosome and discuss its relevance in the clinics.

## 1 The complex role of the BCR signaling in CLL

Among all types of adult leukemia, chronic lymphocytic leukemia (CLL) is the most prevalent lymphoproliferative disease. It is distinguished by the culmination of characteristically 19- and CD5-positive malignant B cells in the bone marrow, blood, spleen and other lymphoid tissues (1–3). CLL displays a highly heterogeneous clinical course, ranging from fast progression with poor outcomes to an indolent course of disease with a good prognosis and regular life expectancy (4). Consistent with this, the selection of a suitable therapy relies on common parameters such as lymphocyte doubling time and the clinical stage of the disease. To date, no single agent to treat CLL was detected (3, 4).

The B cell antigen receptor (BCR) complex consists of an immunoglobulin (Ig) transmembrane protein that is associated with two signal-transmitting subunits, named CD79a (Igα) and CD79b (Igβ) (5). It is critical for normal B cell maturation and survival that the BCR has the capacity to create signals through the Igα/Igβ signaling heterodimer at vital stages during B cell development (6). Moreover, BCR-mediated signaling plays an essential role in the pathogenesis of CLL, as evidenced by multiple sources. First of all, CLL cells retain the surface expression of the BCR, which is a common characteristic among neoplastic B cell malignancies (7). This is based on the fact that malignant B cells derive advantages from the pro-survival signals that are initiated as a result of BCR activation. Second, there is genetic proof across various groups indicating that BCR expression is required for both CLL development (8, 9) and persistence (10, 11). Third, B cell malignancies often exhibit BCR signaling dysregulation and pathways triggered downstream of the BCR were shown to be highly activated in CLL cases (12). BCR-mediated signaling is the primary operating pathway in CLL cells which was identified through gene expression profiling data, particularly in the lymph node (LN) microenvironment, which is thought to support growth and survival of CLL cells (13). Last and most importantly, targeted therapy using BCR pathway inhibitors is a promising approach to treat CLL (14). This is evidenced by the exceptional clinical efficacy of inhibitors targeting BCR signaling, such as ibrutinib (Bruton’s tyrosine kinase (BTK) inhibitor) or idelalisib (Phosphatidylinositol 3-kinase (PI3K) inhibitor), in reversing the CLL disease phenotype suggests an enormous importance of BCR-derived survival signals for CLL cell persistence (15–18).

In more than 30% of cases, patient‐derived CLL cells express similar, or even identical, BCRs with corporate stereotypic features and sequence similarities (19, 20). According to the expressed stereotypic BCR heavy chain, CLL cells are classified into distinct CLL subsets. Each subset displays common genomic abnormalities (21) as well as highly homogeneous clinical and biological properties (22). Altogether, the IGHV-D-J gene recombination pattern and the amino acid constitution of the heavy chain variable complementarity determining region 3 (HCDR3) categorize these stereotyped CLL cases into 19 major subgroups (1, 22, 23). Very recently, a detailed study on BCR stereotypy reported that not only 30% but even 41% of all CLL cases can be categorized into stereotypic subgroups with overall 29 major subsets (20). Most interestingly, the clinical outcome of CLL is determined by the molecular specifics of the stereotyped interactions between BCRs (24). For example, tightly bound and long-lasting BCR-BCR crosslinking interactions in the CLL subset #4 lead to B cell anergy. This is a clinically inactive state which is specifically observed in CLL clones of the subgroup #4 (24, 25).. In contrast, subgroup #2 CLL cases have a more aggressive course of disease due to a low-affinity and rapidly dissolving BCR-BCR interaction, leading to an enhanced signaling activity by the BCR (24).

This significant limitation in the CLL Ig gene repertoire proposes that BCR recognition of a restricted set of antigen epitopes results in the selection and expansion of B cell clones that ultimately enter the pathogenesis of CLL. Epidemiological studies did show that several infectious diseases can be linked to the development of CLL, and CLL-associated Igs react with a variety of viruses or pathogens, suggesting a pathogen-induced CLL development (26–28). In an Eµ-TCL1 mouse study, however, an accelerated development of leukemia could not be observed due to an acute or chronic infection. Preferably, a BCR-mediated autoantigen recognition resulted in the pathogenesis of CLL (8). Interestingly, the murine CLL cells preferred the selection of specific light chains that allowed BCR cross-reaction with a large number of autoantigens (8). This is consistent with the hypothesis, based on evidence obtained in CLL patients, that light chains in combination with defined heavy chains are important for the formation of the leukemic BCR specificity (29, 30). Iacovelli et al. observed that autonomous BCR signaling as well as low-affinity BCR interactions with self-antigens were actively selected during leukemia development in Eµ-TCL1 mice, implying a crucial involvement of these two factors in the pathogenesis of CLL (9). Furthermore, in this model, autoantigen-induced BCR signaling resulted in a more aggressive course of CLL (9). Similarly, a correlation between the response to BCR binding and shorter survival was reported in CLL patient analyses (31). In PtC-reactive Eμ-TCL1 leukemic cells, the response to autoantigen stimulation also resulted in a more aggressive disease (32). Therefore, aberrant autoantigen-induced responses induce an accelerated CLL progression, underlining the great variance in the clinical course of CLL. However, not only low-affinity but also high-affinity BCR-antigen cross-linking cooperates with autonomic BCR-BCR interactions in triggering CLL. For instance, high-affinity binding between three stereotypically mutated CLL subset BCRs and the Fc portion of human IgG was reported, as well as high-affinity binding to the fungal antigen β- (1, 6)-glucan (33, 34).

### 1.1 CLL-associated mutations in the IGHV and IGLV genes

On the basis of the somatic hypermutation (SHM) status in the variable region of the Ig heavy-chain (IGHV) gene, CLL can be categorized into two main types of disease: the unmutated CLL (U-CLL) and the mutated CLL (M-CLL) (35). While in U-CLL cases, the IGHVs show > 98% identity to the germline Ig sequence, the IGHVs of M-CLL show less than 98% homology to the germline sequence. In particular, this IGHV-mutation-based classification represents a strong prognostic marker for CLL. In general, U-CLL cases are associated with a more aggressive form of CLL relative to the mainly indolent course of disease in M-CLL patients, which also experience a longer progression-free survival (PFS) (35, 36). The CLL-Ig repertoire is characterized by the high representation of particularly selected IGHV-coding genes termed IGHV1-69, IGHV3-21, IGHV3-23, IGHV3-7 and IGHV4-34 (37, 38). The IGHV1-69 gene is most frequently selected in the U-CLL group, whereas the IGHV3-21, IGHV3-23, IGHV3-7, and IGHV4-34 genes are typically associated with a high mutational burden (37, 38). Among the CLL subsets expressing stereotyped HCDR3 sequences, Murray et al. observed recurrent amino acid modifications in the IGHV domain, especially in those expressing IGHV4-34 and IGHV3-21 genes, which show unique structures of SHM (39). Since the described mutations are represented in low frequency among non-CLL IGHV domains, they can be considered as CLL-specific (39). CLL research mainly focuses on studying IGHV sequence structures. However, increasing evidence suggests that the Ig light chain (IGLV) sequence has effects on the clinical course and the outcome of CLL as well [reviewed in (40)]. Lately, the IGLV3-21 gene was identified as a prognostic marker for CLL with a poor prognosis, independent of the corresponding heavy-chain (41). Compared to the overall incidence of CLL (7%), increased recurrence of the IGLV3-21 gene (28%) was observed in high-risk CLL cohorts (41). Furthermore, a specific mutant form of the IGLV3-21, the IGLV3-21^G110R^, was highlighted to play an important role in the pathogenesis and prognosis of CLL. This mutation increases the probability of homotypic BCR interactions, resulting in autonomous BCR signaling (42). Thus, IGLV3-21^R110^–expressing CLL cells represent a definite subset of CLL with poor prognosis, irrespective of the IGHV mutational status (42).

The self-activation of CLL-derived BCRs is a significant factor in the development of CLL. This is accomplished through the interaction of CLL-derived BCRs with specific BCR epitopes that are unique to certain subsets. This interaction results in the activation of BCR-mediated pro-survival signaling within CLL cells.

## 2 BCR-mediated autonomous signaling in CLL

Autonomous, antigen-independent BCR signaling was identified as the mechanistic basis of malignant BCR signaling in most of the CLL cases (9, 43). Self-activation of the CLL-derived BCRs is a significant factor in the progression of CLL pathogenesis. This autonomous signaling is induced by the BCRs’ ability to interact with their own defined BCR epitopes that are unique to certain CLL subsets. This interaction results in the activation of BCR-mediated pro-survival signaling within CLL cells (9, 43). This antigen-independent signaling is enabled by an intermolecular cross-link of an oncogenic HCDR3 domain with unique motifs located between the FR2 and FR3 domains within the Ig molecule (43). Each CLL case may acquire specific autoreactive BCRs through certain affinity maturation processes, including the incorporation of distinct SHMs and class-switch recombination (24, 39). Structural analysis of the CLL subset #2 and #4 BCRs revealed the origin of the G110R mutation, which is crucial for homotypic BCR interaction, by a nonsynonymous SHM of the G110 residue in the IGLJ germline segment of the BCR (24). CLL patients carrying the IGLV3-21*01 light chain allele exhibit a higher risk of generating CLL. This specific allele facilitates the acquisition of the malignant G110R mutation, which promotes strong BCR-BCR interaction initiating self-directed BCR signaling (42). Autonomous signaling causes higher basal Calcium (Ca^2+^) signaling and increased activity of signaling factors downstream of the BCR such as BTK, the spleen tyrosine kinase (SYK), and the phosphatidylinositol 3-kinase (PI3K) (44). Most importantly, reversion of the R110 residue into G110 abolishes BCR autonomous signaling (24).

## 3 BCR-mediated downstream signaling in CLL

### 3.1 The BCR signaling subunits Igα and Igβ

The BCR is associated with a signaling heterodimer that consists of two subunits, Igα and Igβ. The Igα/Igβ subunit is required for a proper membrane transport of the Igs for BCR surface expression. Moreover, the mediation of BCR signaling by the Igα/Igβ heterodimer is essential for the maturation, differentiation and survival of B cells (6, 45). The Igα/Igβ subunits are implicated in the BCR complex formation and stabilization. Furthermore, Igα and Igβ facilitate assembly and steadiness of BCR, promote IgM transport to cell surface and increase BCR surface expression levels by regulating its glycosylation (45, 46). In CLL samples, defective glycosylation and subsequent impaired folding of the IgM and CD79a chains leads to impaired BCR assembly as well as reduced surface membrane (sm)IgM expression (47). It was revealed that CLL cells expressing low CD79b protein levels also exhibit reduced expression levels of IgM-BCR complexes. The cytokine IL-4, however, is able to restore CD79b and smIgM expression and is thereby enhancing the activation of BCR-mediated survival signaling in CLL cells (48).

Recently, our group demonstrated that an induced loss of the Igα subunit in CLL cells of a Eµ-TCL1 mouse model, results in an almost complete loss of the diseased cells, indicating a crucial involvement of the BCR in the persistence of CLL cells (10).

**Figure 1:**
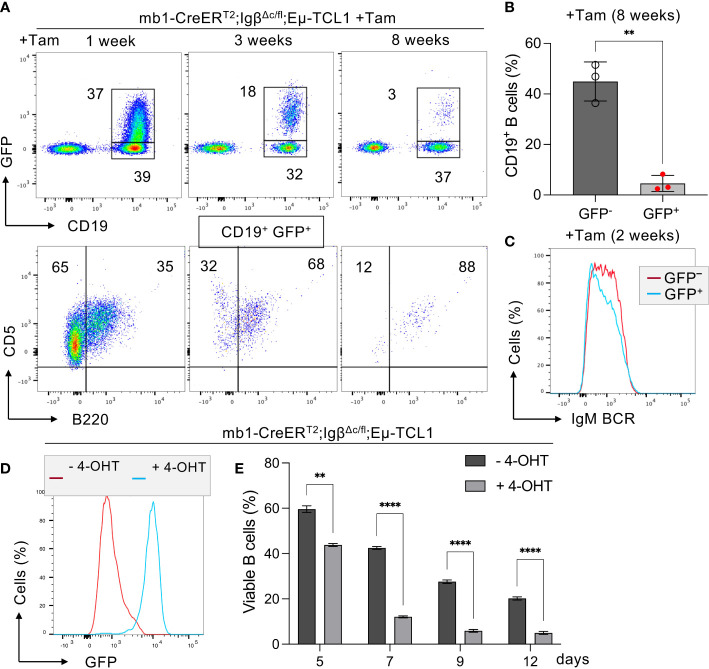
Igβ signaling tail deficiency in Eµ-TCL1 mice leads to CLL cell reduction. **(A)** Flow cytometry assessment was performed on B cells from the peripheral blood (PBL) of diseased mb1-CreER^T2^;Igβ^Δc/fl^;Eµ-TCL1 mice, 1 week (left), 3 weeks (middle), plus 8 weeks (right) following the initiation of Tamoxifen (Tam) treatment. The dot plots of the anti-CD19 versus GFP staining are shown. The CD19^+^GFP^+^ gated region indicates Igβ tail deficient B cells, while the CD19^+^GFP^-^ gated region marks the Igβ tail sufficient B cell population. The B220 vs CD5 staining of the CD19^+^GFP^+^ gated cells is depicted below. The B220^-^CD5^+^ population represents diseased CLL cells, while the B220^+^CD5^+^ gated region exhibits healthy cells. The average relative frequency of the cells within the gate is indicated by the numbers in the dot plots. The data is presentable for three independent mouse analyses. **(B)** Eight weeks after administering Tam treatment to mb1CreER^T2^;Igβ^Δc/fl^;Eµ-TCL1 mice, the percentage of B cells in the CD19^+^GFP^+^ and CD19^+^GFP^−^ B cell populations was quantified. The graphs display the respective average percentage of B cells ± SEM, while p-values were determined by a Student’s t-test (two-tailed; ** p < 0.01). The cell count for each group consists of data from three mice. **(C)** After two weeks of Tam treatment, the fluorescence intensity of IgM BCR expression in CD19^+^GFP^−^ (red) or CD19^+^GFP^+^ (blue) B cells of mb1-CreER^T2^;Igβ^Δc/fl^;Eµ-TCL1 CLL mice was determined. The results presented in the histogram are representative of three self-contained experiments. **(D)** Flow cytometry was used to analyze the expression of GFP in mb1-CreER^T2^;Igβ^Δc/fl^;Eµ-TCL1 CLL cells that were either treated with 4-OHT *in vitro* (+4-OHT blue) or kept untreated (-4-OHT red) for 5 days (5d). The fluorescence intensity of CD19^+^ B cells was determined and indicated in histograms. The data is presentable of three self-sufficient experiments. **(E)** The survival of B cells in mb1-CreER^T2^;Igβ^Δc/fl^;Eµ-TCL1 CLL cells was statistically analyzed on day 5, 7, 9, and 12 following *in vitro* 4-OHT treatment (+4-OHT; light grey). The control remained without treatment (-4-OHT; dark grey). The graphs display the mean ± SEM and p-values were determined by the Student’s t-test (two-tailed; **** p < 0.0001; ** p < 0.01). The results of three independent analyses are presented.

Similarly, we could show that Tam-induced deletion of the intracellular Igβ signaling domain in isolated CLL B cells of mb1-CreER^T2^;Igβ^Δc/fl^;Eµ-TCL1 mice leads to a significant CLL cell regression within 8 weeks (**Figures 1A, B**). In these mice, efficient deletion of the Igβ-encoding gene could be monitored by an induced GFP expression (49) (**Figure 1D**). GFP^+^ cells with Igβ-tail-deficiency maintained IgM BCR surface expression (**Figure 1C**) whereas their viability *in vivo* and *in vitro* was reduced (**Figures 1A, E**). This indicates that the survival, as well as the progression of CLL, depends on the functionality of BCR to generate signals via the Igα/Igβ heterodimer, making it an essential factor.

**Figure 2:**
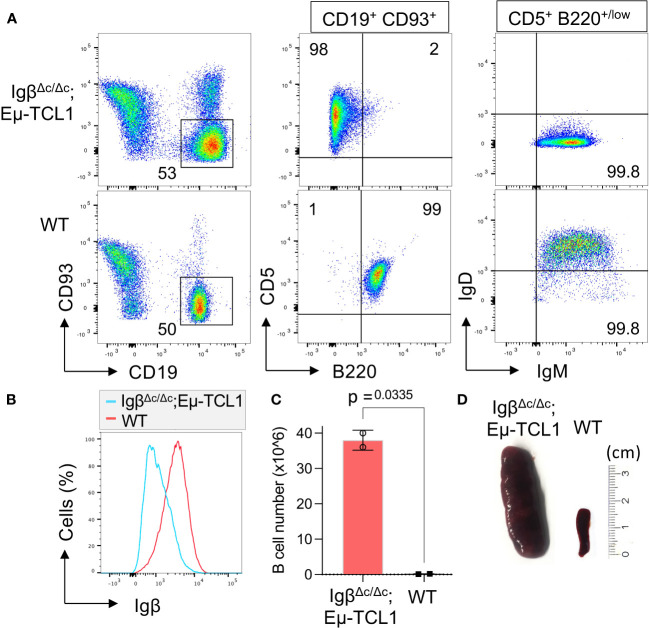
Igβ-tail deficiency in a Eµ-TCL1 mouse model results in CLL development. **(A)** Flow cytometry analysis was conducted on B cells isolated from the spleen of 14-month-old Igβ^Δc/Δc^;Eμ-TCL1 mice and WT control mice. The dot plot depicts the B220 vs CD5 staining of CD19^+^CD93^−^ gated mature B cells, and the IgM vs IgD staining of CD5^+^B220^low^ CLL cells of Igβ^Δc/Δc^;Eμ-TCL1 mice or on normal CD5^−^ B220^+^ B cells of WT mice. The data presented is representative of three self-contained mouse analyses. **(B)** Flow cytometry was used to analyze isolated splenic B cells from Igβ^Δc/Δc^;Eμ-TCL1 mice and WT mice. The fluorescent intensity of Igβ expression is represented in a histogram. **(C)** The absolute number of B cells in the peritoneal cavity of 14-month-old Igβ^Δc/Δc^;Eμ-TCL1 mice and the control mice of the same age were quantified. The graphs represent the average count ± SEM. A two-tailed Student’s t-test was conducted to obtain the p-values. The cell count for each group comprises two mice. **(D)** The images show the spleen of a mb1- Igβ^Δc/Δc^;Eμ-TCL1 mouse and a WT mouse.

**Figure 3:**
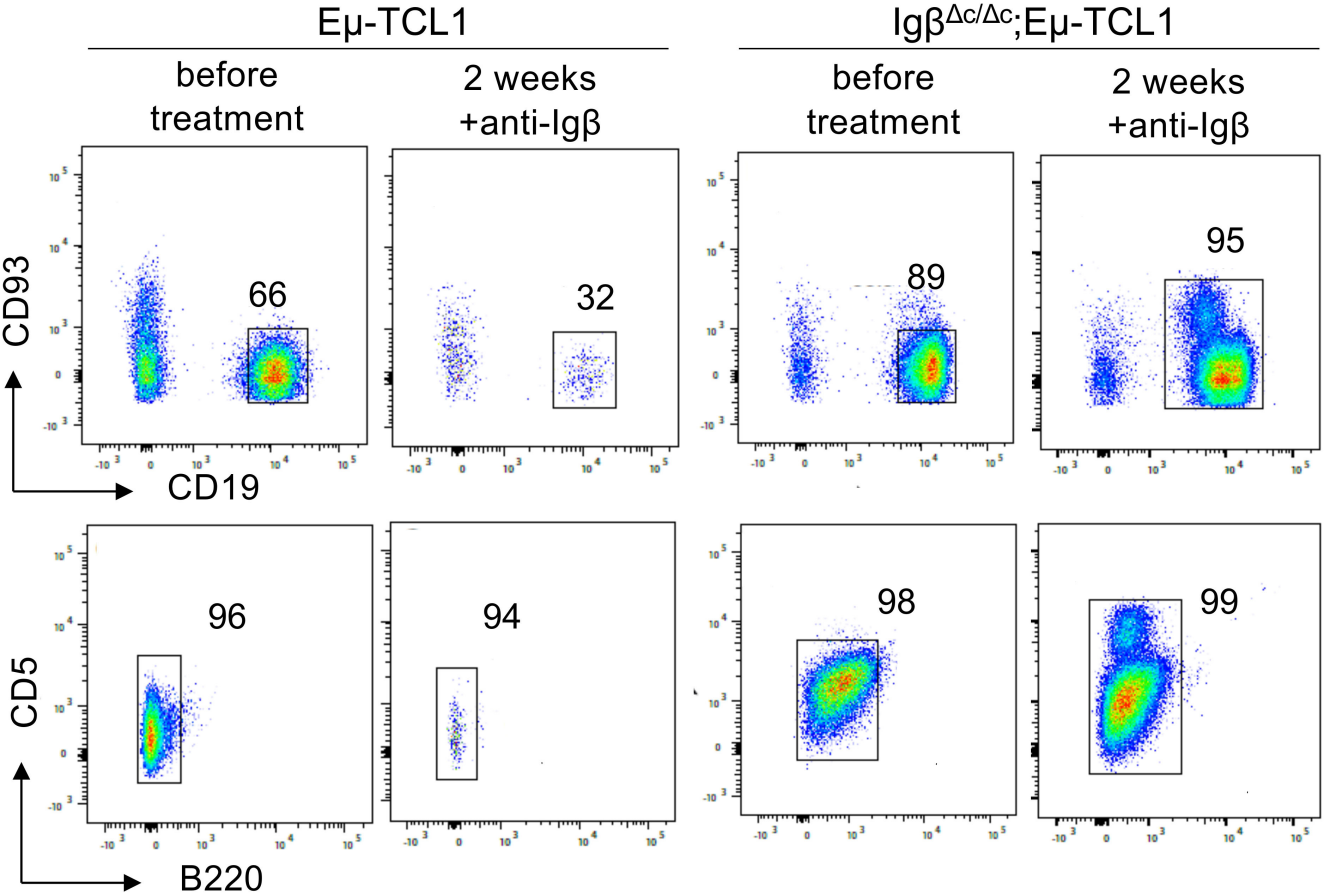
Anti-Igβ treatment does not affect the progression of CLL cells lacking Igβ-tail. CLL cell survival in Eµ-TCL1 and Igβ^Δc/Δc^;Eμ-TCL1 mice was analyzed using flow cytometry one day before and two weeks after administering an anti-Igβ antibody. The dot plot illustrates the staining of anti-B220 vs anti-CD5 mature B cells after gating on the CD19^+^CD93^−^ cell population. CLL cells can be identified by expression of the characteristic markers CD19^+^CD93^−^CD5^+^B220^low^.

Furthermore, we were interested if Igβ signaling tail is essential for CLL progression. Thus, our group tested whether B cells with a constitutive loss of the Igβ signaling tail in the early pro B cell stage are able to develop CLL in an Eµ-TCL1 mouse model. Indeed, constitutive deletion of the Igβ signaling tail in B cells resulted in CLL outbreak of the Igβ^Δc/Δc^;Eμ-TCL1 mice at an age of 12-14 months. The development of the disease was indicated by the accumulation of an increased number of CD5^+^B220^low^ CLL B cells in the spleen of the mouse in combination with splenomegaly (**Figures 2A, C, D**). Efficient deletion of the Igβ tail was validated by flow cytometry (**Figure 2B**). The malignant transformation of B cells in Igβ^Δc/Δc^;Eμ-TCL1 mice despite their Igβ-tail deficiency indicates that expression of the Igβ-tail is not essential for the pathogenesis and persistence of CLL. In addition, CLL cells of Igβ^Δc/Δc^;Eμ-TCL1 mice were not susceptible to anti-Igβ antibody treatment compared to CLL cells that originated from conventional Eµ-TCL1 mice (**Figure 3**) indicating that the CLL cells survive independently of Igβ-tail signaling. So, it is possible that the CLL cells found a way to circumvent Igβ-tail deficiency via deregulation of specific BCR-regulated pathways. However, the susceptibility of Igβ-tail sufficient CLL cells to anti-Igβ antibody treatment suggests a potential clinical efficacy of anti-Igβ antibodies in CLL treatment. However, a clinical phase I trial of polatuzumab vedotin, an anti-Igβ antibody fused to a microtubule-disrupting drug named monomethyl auristatin E, did not show any clinical responses in CLL (50). The missing effect is probably caused by the low or absent Igβ surface expression levels observed in CLL patients. This also explains the lack of Igβ-targeting chimeric antigen receptor T cell studies in CLL therapy, although they show high efficacy in other B cell lymphomas, such as the diffuse large B cell lymphoma (DLBCL) (51). Interestingly, mutations in the extracellular and transmembrane regions of the Igβ-encoding gene B29 were detected in CLL patients, which show aberrant BCR signaling (52). However, no mutations of the Igα-encoding gene were observed (45). It might be possible that the mutations in the B29 gene play a role in CLL oncogenesis. So far, only the Igβ subunit of the BCR complex was targeted for therapy of B cell diseases. However, recently a synergistic potential of combined Igα-targeted and Igβ-targeted therapy of B cell leukemia was observed. Showing high antitumor activity in DLBCL, this method may also be an option in the future treatment of CLL (53).

The Igα/Igβ heterodimer forms the center of the intricate BCR signaling network with essential functional implications for both normal and leukemic CLL cells. Overall, we could show that the expression of a functional BCR complex is essential for the survival of CLL cells (**Figure 1**), CLL development, however, could take place despite restricted BCR signaling in Igβ signaling tail deficient B cells (**Figure 2**). It might be interesting to identify to what extent the detected mutations of Igβ modulate BCR signaling or play a role in CLL leukemogenesis. Studies focusing on the mechanism of Igα/Igβ ubiquitination and glycosylation in CLL may also uncover another layer of BCR signal regulation.

### 3.2 The Src family kinase LYN

**Figure 4:**
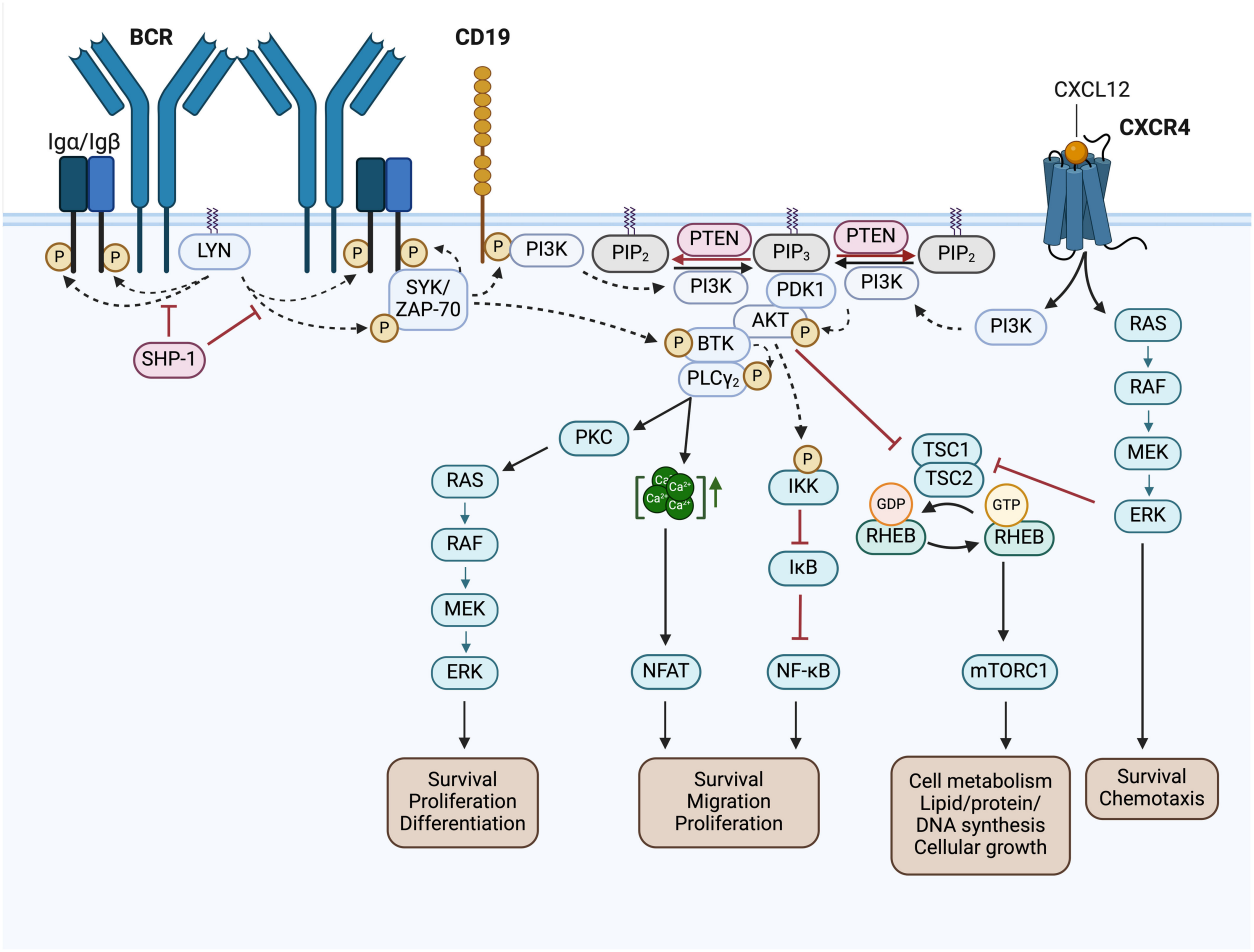
BCR signaling pathway and BCR-associated CXCR4 signaling in CLL. Activated Src family kinases (SFKs), such as LYN, stimulate the phosphorylation of the immunoreceptor tyrosine-based activation motifs (ITAMs) located in the cytoplasmic Igα/Igβ signaling subunit of the BCR. This results in the recruitment and activation of SH2 domain-containing effectors, like SYK and ZAP-70, which phosphorylate BTK and CD19. SHP-1 inhibits the phosphorylation of the Igα ITAMs and SYK. Phosphorylated CD19 recruits the PI3K to the cell membrane, where it phosphorylates PIP_2_ to generate PIP_3_. Thereby, PI3K creates an essential docking platform for PH domain-containing signaling factors, such as PDK1, BTK and AKT. Binding to PIP_3_ results in membrane recruitment and activation of PDK1, BTK and AKT, which mediate the initiation of several BCR downstream signaling cascades, such as RAS/RAF/MEK/ERK signaling, NFAT, NF-κB and mTORC1 signaling. The phosphatase PTEN represses PI3K signaling by PIP_3_ dephosphorylation, generating PIP_2_. NFAT is activated by increased cytoplasmic Ca^2+^ concentrations, which are induced by PLCγ. NF-κB is retained in an inactive state by the inhibitor IκB. Phosphorylation of IKK leads to IκBs phosphorylation and degradation, finally resulting in NF-κB activation. TSC1/2 inhibits RHEB GTPase activity, which is required to induce mTORC1 activation. AKT- or ERK-mediated inhibition of TSC2, results in mTORC1 activation. In addition, binding of CXCL12 to CXCR4 induces CLL cell migration, survival and chemotaxis via the activation of the downstream signaling pathways MAPK/ERK, PI3K/AKT, PLCγ/Ca^2+^ and NF-κB. This figure was created with BioRender.com. BCR, B cell receptor; LYN, LCK/YES novel kinase; SYK, spleen tyrosine kinase; ZAP-70, CD3ζ-chain-associated protein of 70 kDa; SHP-1, Src homology region 2 domain-containing phosphatase-1; PI3K, phosphatidylinositol 3-kinase; PTEN, phosphatase and tensin homolog; PIP_2_, phosphatidylinositol-4,5-bisphosphate; PIP_3_, phosphatidylinositol-3,4,5-triphosphate; PDK1, 3-phosphoinositide-dependent protein kinase 1; PKB/AKT, protein kinase B; BTK, Bruton’s tyrosine kinase; PLCγ, phospholipase Cγ; PKC, protein kinase C; CXCL12, chemokine C-X-C motif ligand 12; CXCR4, C-X-C motif chemokine receptor; RAS, RAF, Rat sarcoma protein family; MEK; ERK, extracellular-signal-regulated kinase; IKK, IκB kinase complex; IκB, inhibitor of nuclear factor κB; NF-κB, nuclear factor kappa-light-chain-enhancer of activated B cells; NFAT, nuclear factor of activated T-cells; TSC1/2, tuberous sclerosis complex 1/2; RHEB, Ras homolog enriched in brain; mTOR complex mTORC1, mechanistic target of rapamycin; Ca^2+^, Calcium-Ion; GTP, Guanosine-5′-triphosphate; GDP, Guanosindiphosphat; P, phosphorylation.

BCR signaling is initiated by the enzymatic activation of the receptor-associated Src family kinases (SFKs), like LYN, FYN, LCK and BLK. Activated SFKs stimulate the phosphorylation of the immunoreceptor tyrosine-based activation motifs (ITAMs) located in the cytoplasmic Igα/Igβ signaling subunit of the BCR. This results in the recruitment and activation of tandem Src homology 2 (SH2) domain-containing effectors, like SYK, which cause the initiation of several BCR downstream signaling pathways (**Figure 4**) (54, 55). The LCK/YES novel kinase (LYN) is distinct from other SFKs in its additional capability to induce phosphorylation of the immunoreceptor tyrosine-based inhibitory motif (ITIM) of inhibitory surface receptors, which recruit tyrosine phosphatases like SHP-1/2 and PP2A. These phosphatases attenuate the B cell activation response triggered by the BCR (56). SHP-1, for instance, counteracts the phosphorylation of the Igα ITAMs and the BCR signaling effector SYK (57). LYN, as a key regulator of the BCR signaling pathway, is overexpressed in CLL patients, and elevated LYN protein levels correlate with a shorter treatment-free survival (58). The increased activity of the LYN kinase observed in CLL cells is also associated with defects in apoptosis mediated by interactions of LYN with the procaspase-8 (59) or SHP-1 (60). Thus, SHP-1 is also found to be expressed at low levels in CLL cells compared to the expression in normal B cells (60).

Although LYN is well-known to balance BCR signaling, LYN activation seems to be dispensable in the development of CLL, since B cell specific gain- and loss-of-function mutations of LYN showed no significant changes in CLL progression in Eµ-TCL1 mice (61, 62). However, several studies suggest an emerging new role of LYN as a crucial regulator within the CLL tumor microenvironment supporting leukemic cell growth and CLL progression (61, 63, 64). For example, LYN-deficiency in macrophages reduces their capability to support CLL cell survival (61). Furthermore, LYN controls the stromal fibroblast polarization, which was shown to support CLL cell survival and leukemic progression. Genetic *Lyn* deletion in stromal cells, for instance, results in reduced expression of c-JUN. This transcription factor is required to induce Thrombospondin-1 expression, which impairs CLL viability by binding to CD47 (64). Thus, the efficacy of LYN inhibition in CLL is to some extent based on an emerging new function of LYN in regulating the tumor microenvironment and the dialog between leukemic cells and bystander cells.

Targeting LYN *in vitro* using the Src/c-Abl tyrosine kinase inhibitor dasatinib blocks CLL cell proliferation and triggers apoptosis in isolated CLL cells (65). Moreover, as a result of dasatinib treatment, reduced BCR-downstream signaling activation and a block in the anti-apoptotic MCL-1-dependent increase in CLL cell survival was observed (66). However, the clinical LYN-targeting drug dasatinib shows by far less effectiveness in the treatment of CLL patients compared to other BCR pathway inhibitors, that will be described later. In a phase II clinical trial, dasatinib treatment achieved partial responses in 3 out of 15 patients (20%; 90% CI 6-44%), and nodal response in 9 patients (60%), indicating only partial success in a small population of patients with relapsed and refractory CLL (67). These findings question the importance of LYN in CLL development and progression. However, since dasatinib has a wide target spectrum it is not a precise tool for evaluating functional relevance of LYN in CLL. CLL studies using different LYN-targeting inhibitors would offer additional insights into the role of LYN in the treatment of CLL.

### 3.3 The spleen tyrosine kinase family

#### 3.3.1 SYK in ‘tonic’ BCR signaling

The spleen tyrosine kinase is part of the SYK family of cytoplasmic non-receptor tyrosine kinases. It is a key component in the BCR-mediated signal transmission and regulates numerous physiological functions in B cells. Recruitment of SYK to phosphorylated ITAM sequences leads to the phosphorylation of more ITAM tyrosines of adjacent BCRs. This generates a positive feedback loop amplifying BCR signal transduction (57). Moreover, SYK directly phosphorylates and activates BTK and mediates PI3K activation by the phosphorylation of its adaptor CD19 (55). The activation of BCR signaling by SYK is counteracted by the tyrosine phosphatase SHP-1 (**Figure 4**) (57).

Gene expression of SYK along with its downstream signaling pathways is significantly enhanced in CLL cells (68, 69). Interestingly, SYK expression in U-CLL cells is increased compared to leukemic cells harboring mutated IGHV domain genes (69). Additionally, SYK is constitutively phosphorylated on activating tyrosines (68). However, to date, no mutations of SYK were detected in patients with CLL (70). Impaired BCR signaling was associated with CLL progression, making SYK a prospective therapeutic target in treating the disease. Preclinical studies in CLL cell lines displayed an effective block in the BCR signaling mediated basal activity of several pro-survival factors after SYK inhibition. These factors include AKT, the extracellular signal-regulated kinases (ERK), plus the anti-apoptotic factor MCL-1, which causes apoptosis of malignant CLL cells (68). Moreover, SYK was observed to be essential in integrin signaling, α-tubulin phosphorylation and CXCL12-mediated polarization of B lymphocytes (71). Thus, inhibition of SYK activity results in markedly reduced migration of the CLL cells toward CXCL12, a key homing attractor. Furthermore, SYK inhibition reduces the adhesion to VCAM-1, an important stromal integrin ligand, and decreases the secretion of CCL4 and CCL3 in CLL cells (72). This disruption of the interaction between the CLL microenvironment and the surrounding stroma through SYK inhibitors attenuates the integrin-/chemokine-mediated protective stromal survival effects in CLL (72). In addition, SYK inhibitors were shown to abrogate CD40 ligand-induced blastogenesis and CLL cell proliferation but not the proliferation of normal B lymphocytes (73).

##### 3.3.1.1 SYK-targeting inhibitors

In clinical trials, the SYK inhibitors fostamatinib disodium (74), entospletinib (75) and cerdulatinib (76), for example, did show selective CLL growth-inhibitory effects. Fostamatinib is the first reported SYK inhibitor (also known as R788), that is metabolized to R406 *in vivo*. In a Eµ-TCL1 murine CLL model, fostamatinib was found to selectively inhibit the growth of the leukemic B cell population, which resulted in significantly prolonged the animal survival (77). Fostamatinib showed an overall response rate (ORR) of 54.5% [only partial response (PR)] and a median progression-free survival (PFS) of 6.4 months (95% CI, 2.2-7.1) in CLL patients participating in a clinical phase I/II study (74). Entospletinib (GS-9973), like other classes of drugs inhibiting BCR signals, disrupts the cellular interactions with the tumor microenvironment and causes a redistribution of CLL cells, which clinically manifests by LN depletion and transient lymphocytosis (75). In an entospletinib phase II trial, patients with relapsed/refractory (R/R) CLL showed an ORR of 61% (95% CI, 44.5%-75.8%; all partial responses) and a median PFS of 13.8 months (95% CI, 7.7 months to not reached) (**Table 1**) (75). However, in another phase II study later on with R/R CLL patients that received prior treatment with a BCR inhibitor, the ORR of entospletinib was only 32.7% (95% CI, 21.7-45.3%) with a PFS of 5.6 months (95% CI, 3.7-8.3) (**Table 1**) (78). Recently, a phase I/II clinical trial of the CD20-targeting drug obinutuzumab in combination with entospletinib in patients with R/R CLL was completed with a promising outcome. Among the 21 R/R-CLL participants that received ≥1 prior therapy, the ORR was 67% (95% CI, 43-85%) with 14% (95% CI, 3-36%) achieving a complete response (CR), and 53% a partial response (PR). Median PFS was 27.5 months (95% CI, 16 months – not reached) (**Table 1**) (79). Cerdulatinib (PRT062070), an inhibitor of SYK and the Janus kinases JAK1/3, inhibits BCR- along with IL4-mediated signaling in CLL cells and reduces CCL3/CCL4 production to overcome stromal support (96). Moreover, cerdulatinib effectively induces apoptosis and inhibits the proliferation of ibrutinib-resistant CLL cells protected by the tumor microenvironment (97). Cerdulatinib treatment in a phase I study resulted in a restricted response in 3 out of 8 R/R CLL patients, demonstrating promising antitumor activity (76). A phase IIa study (NCT01994382) continued to assess cerdulatinib’s tolerability and efficacy in patients affected by R/R B cell lymphomas, including CLL, and displayed an ORR of 61% (80).

**Table 1:** Outcome of selected clinical trials using BCR signaling targeting inhibitors.

Target	Agent	Phase	Patient [*n*]	Response	Trial information
LYN	Dasatinib	II	15	PR 20% (90% CI, 6-44%), nodal response 60% (13% CR; 47% PR)	Relapsed fludarabine-treated CLL patients, 73% with high-risk del(11q) or del(17p) (67)
SYK	Fostamatinib	I/II	11	mPFS 6.4 months (95% CI, 2.2-7.1), ORR 54.5% (PR)	R/R CLL patients (74)
	Entospletinib	II	49	ORR 32.7% (95% CI, 21.7-45.3%), NR 48.8%, mPFS 5.6 months (95% CI, 3.7-8.3)	R/R CLL patients, which received prior therapy with a BCR signaling inhibitor (78)
	Entospletinib	II	41	ORR 61% (95% CI, 44.5%-75.8%), only PRs, mPFS 13.8 months (95% CI, 7.7 months - not reached)	R/R CLL patients (75)
	Entospletinib + Obinutuzumab	I/II	21	ORR 67% (95% CI, 43-85%), CR 14% (95% CI, 3-36%), PR 53%, mPFS 27.5 months (95% CI, 16 months - not reached)	R/R CLL patients that received ≥1 prior therapy (79)
	Cerdulatinib	IIa	28	ORR 61%	R/R CLL patients, median No. of 3 prior therapies (80)
PI3K	Idelalisib	I	54	ORR 72% (95% CI, 58.4%-83.5%), PR 39%, PRwL 33%, mPFS 15.8 months	R/R CLL patients, median No. of 5 prior therapies, unmutated IGHV 91%, del (17p)/mutTP53 24% (81).
	Idelalisib + Rituximab	III	110	ORR 85.5% (95% CI, 77.5-91.5%), CR 0.9%, PR 84.5%, mPFS 20.3 months (95% CI, 17.3 to 26.3 months), OS 40.6 months (95% CI, 28.5-57.3 months)	R/R CLL patients, median No. of 3 prior therapies, unmutated IGHV 83.6%, del(17p)/mutTP53 43.2% (17)
	Idelalisib + Rituximab + Bendamustine	III	207	ORR 70% (95% CI, 63-76%), CR 1.4%, PR 68.6%, mPFS 20.8 months (95% CI, 16.6-26.4 months; HR = 0.33)	R/R CLL patients, unmutated IGHV 84%, del(17p)/mutTP53 33% (82).
	Duvelisib	III	160	ORR 74%, CR 0.6%, PR 72.5%, PRwL 0.6%, mPFS 13.3 months (HR = 0.52)	R/R CLL patients, median No. of 2 prior therapies, del(17p)/mutTP53 19% (18).
BTK	Ibrutinib	Ib/II	31	ORR 87%, CR 35%, PR 45%, PRwL 6%, mPFS NR	First-line treated patients, unmutated IGHV 48%, del(17p) 6% (16).
	Ibrutinib	Ib/II	101	ORR 89%, CR 10% PR 76%, PRwL 3%, mPFS 52 months (95% CI, 38–70)	R/R CLL patients, median No. of 4 prior therapies, unmutated IGHV 78%, del(17p) 34% (16)
	Ibrutinib + Venetoclax	III	260	ORR 95.4% (95% CI, 92.1-97.6%), CR 71.5% (95% CI, 65.6%, 76.9%), mPFS NR, 3-year PFS rate 97.2% (HR = 0.13; 95% CI, 0.07-0.24)	First-line treatment, treatment-naive CLL, unmutated IGHV 56.9%, del(11q) 20.6%, del(13q) 31.4% (83).
	Acalabrutinib	I/II	99	ORR 97%, CR 7%, PR 90%, mPFS NR, estimated 48-month PFS rate 96% (95% CI, 89-98%)	TN CLL, unmutated IGHV 62%, mutTP53 18%, del(17p) 10% (84).
	Acalabrutinib	Ib/II	134	ORR 94% (95% CI, 89-97%), CR 4%, PR 84%, PRwL 6%, mPFS NR, estimated 45-month PFS 62% (95% CI, 51-71%)	R/R CLL patients, median No. of 2 prior therapies, unmutated IGHV 73%, del(17p) 23%, del(11q) 18% (85).
	Acalabrutinib	II	60	ORR 78% (95% CI, 66-88%), CR 8%, PR 65%, PRwL 5%, mPFS NR, 36-month PFS 58% (95% CI, 42-71%) and OS rate 78% (95% CI, 65-87%)	ibrutinib-intolerant R/R CLL patients, median No. of 2 prior therapies, del(17p) 28% (86)
	Zanubrutinib	III	109	ORR 94.5%, CR 3.7%, PR 87.2%, PRwL 3.7%, mPFS NR, 18-month PFS 88.6% (95% CI: 79.0–94.0) and OS rate 95.1% (95% CI: 88.4–98.0)	treatment-naïve CLL with del(17p) 100% (87).
	Zanubrutinib	I/II	192	ORR 91%, CR 8%, PR 83%, mPFS 61.4 (95% CI, 40.5 – NR) months, 36-month PFS 72.9% and OS 80%	R/R CLL patients, unmutated IGHV 40.6%, del(17p) 14.6%, mutTP53 50%, del(11q) 22% (88).
	Zanubrutinib + Obinutuzumab	Ib	45	ORR (TN) 100%, 30% CR, 70% PR; ORR (R/R) 92%, 28% CR, 64% PR; mPFS NR	R/R CLL patients (56%), TN CLL (44%) (89)
	Tirabrutinib	I	28	ORR 96%, estimated mPFS 38.5 months, median OS 44.9 months	R/R CLL patients, median No. of 4 prior therapies (not BTKi), unmutated IGHV 84%, mutTP53 52%, del(17p) 36% (90, 91).
	Tirabrutinib + Idelalisib + Obinutuzumab	II	30	ORR 93.3% (95% CI, 80.5-98.8%), CR 6.7%, PR 86.7, mPFS NR (90% CI, 22.3-NR), 24-month PFS 80.6% (90% CI, 41.1%-94.9%), and OS 96.7% (90% CI, 83.9%-99.3%)	R/R CLL, unmutated IGHV 63%, del(17p)/mutTP53 33% (92).
	Pirtobrutinib	I/II	247	ORR 82.2% (95% CI, 76.8-86.7), CR 1,6%, PR 71,7%, PRwL 8.9%, mPFS 22.1 months (95% CI, 19.6-27.4)	R/R CLL patients, median No. of 3 prior therapies (all BTKi), mutBTK^C481^ 37.8%, mutPLCγ_2_ 8%, del(17p)/mutTP53 46.6% (93)
	Pirtobrutinib	I/II	100	ORR 79.0% (95% CI, 69.7-86.5%), PR 70%, PRwL 9%, mPFS 16.8 months (95% CI, 13.2-18.7)	R/R CLL patients, median No. of 3 prior therapies, 100% prior BTKi and BCL2i treatment (93)
	Nembtabrutinib	I	29	ORR 75% (PR), mPFS NR (95% CI, 16.7 months – NR) for patients treated at 65mg once daily (n = 8)	R/R CLL, median No. of 5 prior therapies, mutBTK^C481^ 82.8%, mutTP53 38%, del(17p) 24% (94).
	Nembtabrutinib	I/II	57	ORR 56% (95% CI, 42 – 69%), CR 3.5%, PR 26.3% PRwL 26.3%, mPFS 26.3 months (95% CI, 10.1 - NR)	R/R CLL, median No. of 4 prior therapies (cBTKi 95%, cBTKi + BCL2i 42%, mutBTK^C481^ 65%, del(17p) 33%, mutTP53 32% (95).
	Vecabrutinib	Ib	30	(1/30) PR 0.4%, (11/30) stable disease 37%.	R/R CLL, median No. of 4 prior therapies, mutBTK^C481^ 55.2%, del(17p)/mutTP53 73.3%

ORR, overall response rate; CR, complete remission; PR, partial remission/response; PRwL, partial response with lymphocytosis; mPFS, median progression-free survival; OS, median overall survival; NR, not reached; CI, confidence interval; HR, hazard ratio; R/R, relapsed refractory; TN, treatment-naïve.

Altogether, by effectively blocking BCR downstream signaling activity and by disrupting the protective interactions with the CLL microenvironment, SYK inhibition represents a promising strategy for treating R/R-CLL. The best SYK-targeting efficacy in CLL treatment was reached by the SYK inhibitor entospletinib in combination with the CD20-targeting drug obinutuzumab.

#### 3.3.2 ZAP-70 - a prognostic marker in CLL

The two SYK family members SYK and the CD3ζ-chain-associated protein of 70 kDa (ZAP-70) are structurally homologous and also have a similar functional role in initiating proximal receptor signaling with slight differences, for example in their dependency on Src-family kinases for their catalytic activation (98). Although ZAP-70 was first solely described in T cells, it is also expressed partially in B-CLL cases and was found in other B cell malignancies (99). Although the activation of ZAP-70 was observed to be not quite efficient in CLL cells, the kinase is able to enhance BCR signaling independent of the phosphorylation status of its activating tyrosines (100). It was shown that ZAP-70 constitutively promotes gene expression, protein synthesis as well as microenvironment interactions in CLL cells. ZAP-70 mediated tonic BCR signaling induces an enhanced transcription of the genes coding for the proto-oncogene MYC and the T cell chemokines CCL3/CCL4. These chemokines stimulate the recruitment of T cells into proliferation centers, where they provide a supportive microenvironment. Thereby, ZAP-70 improves CLL cell fitness to survive and proliferate and further drives disease progression (101). This tonic BCR signaling is solely present in U-CLL patients and relies on the ability of ZAP-70 to stimulate the activation of AKT (102). Aberrant ZAP-70 expression in CLL correlates with an unmutated IGHV gene status the selection of unmutated IGHV region genes (103), the expression of a typically self-reactive BCR (43) and a poor clinical outcome (102). In contrast, B CLL cells lacking ZAP-70 expression are mainly anergic, lose BCR responsiveness, and generally result in a more indolent course of disease (104). Hence, ZAP-70 is used as a reliable prognostic marker for CLL (103). Recent findings suggest that ZAP-70 largely suppresses SYK-mediated BCR-signaling and rather redirects BCR-SYK-mediated signaling from Ca^2+^-NFAT signaling toward the activation of the PI3K signaling pathway (105). This helps B cell clones to escape the NFAT-induced anergic state followed by negative selection that would typically cause elimination of autoreactive or pre-oncogenic B cells. Thus, expression of ZAP-70 in B cells allows sustained signaling induced by autoreactive BCRs, thereby facilitating malignant BCR-mediated B cell transformation (105).

Most interestingly, ZAP-70 not only mediates constitutive BCR signaling, but recently was also found to regulate chemokine-mediated signaling. For example, the CCL19- and CCL21-induced cell migration of U-CLL cells is regulated by the function of ZAP-70 to enhance CCR7 signaling (102). This new data was also presented in the ASH meeting 2023 (102). It provides important new explanations for the enhanced CLL cell fitness in ZAP-70 positive CLL and the more aggressive clinical course of the disease. To what extent this activity of ZAP70 is linked to the expression of unmutated IGHV region genes in CLL still requires clarification.

### 3.4 PI3K/AKT signaling in CLL

The phosphatidylinositol 3-kinase (PI3K) transduces signals from the BCR, chemokine receptors, plus adhesion receptors, thereby promoting the development, survival, chemotaxis as well as the cytoskeletal rearrangement of B cells (54, 106). By generating the lipid phosphatidylinositol-3,4,5-triphosphate (PIP_3_), PI3K creates an essential docking platform for PH domain-containing signaling factors, such as BTK, the 3-phosphoinositide-dependent protein kinase 1 (PDK1), or the protein kinase B (PKB, also known as AKT). Binding to PIP_3_ results in membrane recruitment and activation of the named signaling factors, which mediate the initiation of several BCR downstream signaling cascades (**Figure 4**) (12, 55). BCR-dependent signaling via the PI3K-AKT-axis is believed to provide the essential “tonic signal”, that is required for malignant transformation and progression of CLL cells (107). Recently, AKT was found to be overactivated in high-risk CLL and in more than 50% of CLL patients having Richter’s transformation (RT), a highly aggressive form of lymphoma that is developed by 2 – 10% of patients during the clinical progression of CLL (108). Moreover, Kohlhaas et al. identified constitutive AKT activation as a driver of CLL to initiate RT through enhanced Notch signaling of the RT CLL cells with the T cells of their tumor microenvironment (108). The phosphatase and tensin homolog (PTEN) is a tumor suppressor that antagonizes PI3K/AKT signaling. PTEN represses PI3K signaling by PIP_3_ dephosphorylation, which leads to cell cycle arrest as well as apoptosis (12, 54). In CLL patients, PTEN expression was shown to be downmodulated. Furthermore, genetic deletion of *Pten* results in significantly accelerated CLL development in Eµ-TCL1 mice, which underlines its crucial involvement in malignant transformation (10). In line with this, allelic variances in the *Pten* gene-containing locus 10q23.3 could be identified in CLL patients as well as a total loss of heterozygosity. However, no direct genetic *Pten* mutations were found (109). Furthermore, a decreased PTEN expression is associated with a poor CLL prognosis (110), indicating an essential role of PTEN downregulation in the leukemogenesis and progression of CLL.

#### 3.4.1 PI3K-targeting inhibitors

The PI3K is categorized into three different classes (I, II, III). The PI3Ks of class I can be subdivided into four isoforms: PI3Kα, PI3Kβ, PI3Kγ, and PI3Kδ. The isoforms PI3Kγ plus PI3Kδ are expressed in CLL cells and have distinct important functions in regulating BCR signaling, cell migration as well as CLL cell adhesion to stromal cells (111). Idelalisib, a selective PI3Kδ inhibitor, suppresses BCR-mediated signaling as well as CLL cell interactions with the protective tumor microenvironment (112). This causes CLL cell mobilization, resulting in transient lymphocytosis and size reduction of the LNs (113). In a phase I clinical study with idelalisib, a consistent decrease of AKT phosphorylation, reduced secretion of stroma-derived factors (CD40L, CCL2, CXCL13, tumor necrosis factor (TNF)-α) as well as CLL-derived chemokines such as CCL3, CCL4, CCL17 and CCL22 could be observed. Moreover, Idelalisib treatment exhibits a beneficial safety profile and induces a fast and stable disease reduction in R/R CLL patients with poor prognosis, that received a median of 5 prior therapies (81). The ORR of this study reached 72% (95% CI, 58.4%-83.5%), while 39% of the patients had a PR, and 33% showed treatment-induced lymphocytosis. The overall median PFS was 15.8 months, but 32 months with the higher (now recommended) dose of ≥150 mg (81). In patients with R/R CLL, combined therapy of idelalisib and rituximab (a CD20-tageting antibody, frequently used in CLL therapy) results in a higher median overall survival (OS) compared to rituximab therapy alone (17, 114). The OS was 40.6 months (95% CI, 28.5 - 57.3 months) and 34.6 months (95% CI, 16.0 months – not reached (NR)) for idelalisib-rituximab-treated and placebo-rituximab-treated patients, respectively (more data in **Table 1**) (17). However, relative to the placebo group, idelalisib increased the incidence of grade ≥ 3 diarrhea, grade ≥ 3 colitis and grade ≥ 3 pneumonitis to 16.4%, 8.2% and 6.4%, respectively (17). A different combined therapy of the chemotherapy drug bendamustine, rituximab, and idelalisib indicates improved the median PFS relative to bendamustine-rituximab combined treatment in R/R CLL patients (PFS 20.8 (95% CI, 16.6 – 26.4%) vs 11.1 (8.9 – 11.1%) months; hazard ratio (HR) = 0.33 (95% CI, 0.25 – 0.44%). For additional results see **Table 1**. However, a higher risk of infection and generally higher incidence of serious adverse events were observed in the idelalisib-treated group (82). By now, some novel PI3K inhibitors have been developed, including copanlisib (115), duvelisib (116), and umbralisib (117). Based on promising results in a phase III trial (ORR 74%, mPFS 13.3 months (HR = 0.52)), the PI3Kδ and PI3Kγ dual inhibitor duvelisib was approved by the FDA for the treatment of R/R CLL in the year 2018 (18).

Despite the striking success of PI3Kδ inhibitors in CLL therapy, development of resistance upon idelalisib treatment was observed in several patients (81, 114). Recently, hyperactivated insulin-like growth factor1 receptor (IGF1R) signaling was described as a possible mechanism of PI3Kδ inhibitor resistant CLL cells, suggesting IGF1R-targeted treatment as an effective strategy to overcome PI3Kδ inhibitor resistance (118, 119).

In general, the PI3K-AKT signaling axis represents a promising therapeutic target providing an alternative strategy in the treatment of high-risk R/R-CLL. According to recently published data, especially the patients refractory to prior ibrutinib treatment tend to show a more favorable response to idelalisib therapy (120). However, the increased risk of infection and the higher incidence of serious adverse events observed in combination clinical trials comprising ibrutinib treatment (17, 82) questions the tolerability of this PI3K-targeting drug.

#### 3.4.2 AKT-targeting inhibitors

Another promising drug, named OSU-T315, targets the PI3K-AKT signaling axis in a different way: it specifically prevents AKT activation by blocking AKT membrane recruitment without modifying the activation status of receptor-associated kinases. With the disruption of AKT recruitment to lipid rafts, OSU-T315 targets CLL cell survival and triggers caspase-dependent CLL cell apoptosis. *In vitro*, OSU-T315 evidences potential therapeutic effectiveness in high-risk CLL patients with unmutated IGVH, del(17p13.1) or resistance to ibrutinib. Moreover, treatment with OSU-T315 significantly prolonged the survival of TCL1 mice (121). This AKT-targeting inhibitor presents an outstanding novel mechanism in the therapy of CLL and possibly also other B-cell malignancies. Further investigations in a phase I/II clinical trial would provide interesting insights in the tolerability and efficacy of this agent in the treatment of R/R CLL.

### 3.5 The Bruton’s tyrosine kinase

The Bruton’s tyrosine kinase (BTK), a Tec family kinase, is considered a key regulator of (oncogenic) BCR signaling, critical for the pathogenesis and progression of CLL cells (122). BTK is activated downstream of the BCR via PH domain-mediated membrane recruitment to PIP_3_, followed by phosphorylation, either by SYK or an SFK (123). This results in phospholipase C γ2 (PLCγ_2_) activation, which in turn induces the activation of downstream MAPK signaling pathway and the transcription factor nuclear factor of activated T-cells (NFAT). Thereby, BTK links the BCR to its downstream signaling effectors (**Figure 4**) (55). Due to chronic BCR signaling, most CLL cell clones show increased BTK expression as well as constitutive phosphorylation compared to non-malignant B cells (124–126). Beyond its classical role in mediating BCR signaling, BTK also has some other molecular effects. As a key regulator of CXC-chemokine receptor 4 and 5 signaling, BTK controls B cell migration in response to the so-called homeostatic chemokines CXCL12 and 13, as well as tissue homing, integrin-mediated adhesion, homeostasis or cellular retention in supportive lymphoid niches (127). The survival and relapse of CLL cells are thought to partially depend on the interaction of leukemic cells with their tumor microenvironment along with the LN-resident CLL cells (128). Thus, functional inhibition of BTK in primary CLL cells strongly reduces BCR- plus chemokine-controlled retention of leukemic B cells in their protective tumor microenvironment (129). In addition, BTK was shown to function in monocyte/macrophage cell populations, which represent a relevant component of the CLL tumor microenvironment (130). Altogether, BTK provides a promising therapeutic target.

#### 3.5.1 BTK inhibitor ibrutinib

The BTK inhibitor ibrutinib has revolutionized the treatment of CLL patients. In February 2014, ibrutinib was approved by the FDA and nowadays is preferred as first-line therapy for the majority of CLL patients. In a clinical phase Ib/II study, ibrutinib showed high efficacy both, in first-line treatment settings with an ORR of 87% (CR 35%, PR 45%) and in the treatment of R/R CLL (ORR 89%; CR 10% PR 76%) (16). The median PFS was not reached (95% CI, not estimable (NE)-NE) in CLL patients with first-line treatment and 52 months (95% CI, 38–70) in R/R CLL patients. The estimated PFS rate of 7 years was 83% with first-line treatment and 34% with treatment for R/R CLL (16). Ibrutinib covalently interacts with the active site of BTK at cysteine 481 and thereby prevents the signal transmission to BTK-downstream survival pathways such as mitogen-activated protein kinase (MAPKs), PI3K or nuclear factor-κB signaling (126, 131). This results in reduced CLL cell proliferation and apoptosis (126, 131). Increasing evidence indicates a crucial inhibitory role of ibrutinib on constituents of the CLL microenvironment (132). For example, ibrutinib effectively blocks the secretion of survival factors (such as BAFF, CD40L, IL-4, IL-6, TNF-α) and inhibits fibronectin binding, as well as the cellular interaction with the stroma. Thereby, the dialog of the tumor cells with the microenvironment is interrupted (126). In addition, ibrutinib treatment causes a decrease in CD4^+^ and Th17 T cells together with a diminished expression of activation markers on T cells (132). This may be a side-effect of ibrutinib’s ability to also inhibit other kinases such as the interleukin-2-inducible kinase (ITK). ITK is a BTK homolog that plays a role in the activation of T cells as well as natural killer cells (133). Ibrutinib also inhibits chemokine-mediated cellular migration and reduces the production of BCR-induced chemokines such as CCL3 and CCL4 in CLL cells. This results in early transient lymphocytosis together with a reduction in disease progression (134). In ibrutinib-treated CLL patients, the transient lymphocytosis correlates with a size reduction of the spleen and LNs, followed by a rise of leukemic cells in the blood (135). Ibrutinib is supposed to prevent the interaction between CLL cells and microenvironmental stroma cells that support the propagation, maintenance, as well as the resistance of malignant CLL cells (136, 137). By doing so, ibrutinib initiates CLL cell evasion from protective niches, leading to the apoptosis of CLL cells due to a lack of stromal support (132, 138). The most common primary reasons for CLL patients to discontinue ibrutinib treatment were disease progression (first-line, 6%; R/R, 38%) and adverse events (first-line, 26%; R/R, 23%) (16). Although ibrutinib shows already great efficacy with a high ORR in monotherapy, several ongoing clinical studies are currently aiming to discover a combination therapy that increases the efficacy and tolerability of an ibrutinib-monotherapy in the treatment of CLL. In a recent phase III study, presented at the ASH meeting 2023, a significantly improved response in CLL patients treated with a combination of ibrutinib and the BCL2 inhibitor venetoclax was observed with a 3-year PFS rate of 97.2% and an ORR of 95.4% (95% CI, 92.1-97.6%) (**Table 1**). Severe adverse effects were reported in 51% (83). With these results, the combination of ibrutinib and venetoclax indicates superior clinical efficacy and suggests a strong synergy of BCL2 and BCR-dependent pathways. Consequently, ibrutinib-venetoclax seem to be a promising combination for a successful first-line treatment in combatting CLL.

#### 3.5.2 CLL patient’s resistance to ibrutinib

Despite its huge clinical effectiveness, resistance and/or relapse of CLL in patients receiving ibrutinib therapy was frequently observed. The majority of ibrutinib-resistant CLL patients (~ 85%) acquired mutations in the BTK or PLCγ_2_ expressing genes. Especially, the BTK^C481S^ mutation is frequently described. It disables ibrutinib’s capacity to irreversibly bind BTK, culminating in poor clinical outcomes (139). The R665W and L845F mutations of PLCγ_2_, which were identified in ibrutinib-resistant patients, are hypermorphic and induce BCR signaling independent of the BTK activity (140). Both PLCγ_2_ mutants are highly sensitive to activation via the Rho GTPase RAC2, suggesting an important role of RAC2 in activating PLCγ_2_ in a BTK-independent manner (141). Furthermore, SYK and LYN, respectively, were shown to play a role in inducing mutant PLCγ_2_ activity, since inhibition of either SYK or LYN impairs mutant PLCγ_2_-mediated signaling (142). Another study showed that CLL-specific PLCγ_2_ mutants such as PLCγ_2_^S707Y^ are still responsive to a catalytical inactive BTK variant with reduced sensitivity to covalent BTK inhibitors. This activity of noncatalytic BTK may constitute a primary CLL resistance to active-site BTK inhibitors (143). To overcome ibrutinib resistance in CLL treatment, several second-generation BTK inhibitors were generated and extensively studied to evaluate their tolerability plus efficacy in patients with R/R CLL (reviewed in (144)).

#### 3.5.3 Novel covalent BTK inhibitors

##### 3.5.3.1 Acalabrutinib

Acalabrutinib (ACP-196) also binds irreversibly to the BTK C481 active site, however, with a higher selectivity compared to ibrutinib since it is just weakly interacting with the TEC kinase and shows no inhibition of ITK or EGFR, resulting in less adverse effects (145, 146). Among all new-generation BTK inhibitors, acalabrutinib is presently the most advanced drug in clinical development and demonstrates an impressive ORR of 97% (90% PR; 7% CR) in treatment-naïve CLL. In this phase I/II clinical study, the median PFS of acalabrutinib-treated CLL patients was not reached and the 48-month PFS was estimated to 96% (95% CI, 89-98%) (**Table 1**). Serious adverse events were reported in 38% of the CLL patients (84). In R/R CLL patients, acalabrutinib treatment reached an ORR of 94% (95% CI, 89-97%; 4% CR; 84% PR), and an estimated 45-month PFS of 62% (95% CI, 51-71%) (85). In an ongoing phase II study, acalabrutinib presently showed high efficacy and safety in most R/R CLL patients unable to tolerate ibrutinib with an ORR of 78% (95% CI, 66-88%), and an estimated 36-month PFS rate of 58% (95% CI, 42-71%) (**Table 1**). Related to aclarubicin treatment, severe adverse events were experienced by 17% of the patients (86). Another phase III clinical study compared the efficacy of acalabrutinib relative to idelalisib-rituximab or bendamustine-rituximab treatment in R/R CLL patients. The patients treated with acalabrutinib reached a significantly increased 12-month PFS of 88% (95% CI, 81-92%) compared to idelalisib-rituximab or bendamustine-rituximab treatment (68%; 95% CI, 59-75%) (147).

##### 3.5.3.2 Zanubrutinib

Zanubrutinib (BGB-3111) is another covalent BTK inhibitor that irreversibly binds C481 in the BTK active site. Compared to ibrutinib, zanubrutinib exhibits a higher selectivity. Initial data of clinical studies indicate a beneficial activity and safety profile of zanubrutinib in CLL patients, in monotherapy or combined with obinutuzumab (87, 89). At an average follow-up of 18.2 months in a clinical phase III trial, zanubrutinib therapy in treatment-naïve CLL patients with del(17p) mutation achieved an ORR of 94.5% (CR 3.7%, PR 87.2%, PRwL 3.7%), and the 18-month PFS rate was estimated to 88.6% (95% CI: 79.0–94.0) (**Table 1**) (87). In R/R CLL, the ORR of zanubrutinib was 91% (CR8%, PR 83%), with a median PFS of 61.4 months 61.4 (95% CI, 40.5 – NR), and a 36-month PFS and OS of 72.9% and 80%, respectively. Severe adverse effects were reported in 56.9% (**Table 1**) (88). ORR of the Zanubrutinib plus obinutuzumab combination therapy was 100% (n = 20; 30% CR, 70% PR) in treatment-naïve CLL patients and 92% (n = 23; 28% CR, 64% PR) in patients with R/R CLL. The median follow-up was 29 months, median PFS was not reached and serious adverse events were reported in 49% of the patients (89).

##### 3.5.3.3 Tirabrutinib

Tirabrutinib (ONO/GS-4059) covalently inhibits BTK by preventing Tyr223 auto-phosphorylation. Similar to acalabrutinib and zanubrutinib, tirabrutinib was well tolerated in a first ongoing phase I clinical evaluation in patients with R/R CLL, showing an estimative median PFS of 38.5 and 44.9 months overall survival (90). Like in the other BTK inhibitors, a large number of patients (82%) exhibit transient CLL cell lymphocytosis (91). Two phase II trials in R/R CLL patients currently assesses the efficacy and safety of tirabrutinib in combination with entospletinib or idelalisib, without or with the addition of obinutuzumab (NCT02983617 and NCT02968563). Initial data shows high efficacy and tolerability in relapsed CLL patients treated with a combination of tirabrutinib, idelalisib and obinutuzumab with an ORR of 93.3% (95% CI, 80.5-98.8%), and a 24-month PFS and OS of 80.6% (90% CI, 41.1%–94.9%) and 96.7% (90% CI, 83.9%–99.3%), respectively. Serious treatment-emerged adverse events were experienced in 36.7% (92).

#### 3.5.4 Novel non-covalent BTK inhibitors

Noncovalent (reversible) BTK inhibitors differ from the previously mentioned compounds in noncovalently binding BTK, resulting in selective inhibitory effects regardless of a C481S BTK mutation (148). Noncovalent BTK-inhibitory drugs were generated in order to successfully improve the treatment in R/R CLL patients with BTK-inhibitor resistance bearing a BTK C481S mutation. In preclinical trials, the non-covalent BTK inhibitors pirtobrutinib (LOXO-305) (149), nemtabrutinib (ARQ-351) (150), vecabrutinib (SNS-062) (151), and fenebrutinib (GDC-0853) (152), inhibited BCR signaling in BTK C481 mutant cells and/or in animal models.

##### 3.5.4.1 Pirtobrutinib

The non-covalent, orally available, BTK inhibitor Pirtobrutinib (LOXO-305) reversibly blocks the ATP binding site on BTK. It is highly selective with a more than 300-fold selectivity for BTK in 98% of tested kinases (153). Recently, a phase I/II clinical study revealed promising effectiveness of pirtobrutinib in the therapy of patients with heavily pretreated CLL. Patients that received prior BTK inhibitor treatment achieved an ORR of 82.2% (95% CI, 76.8-86.7; CR 1,6%, PR 71,7%, PRwL 8.9%), with a median PFS of 22.1 months (95% CI, 19.6-27.4). An ORR of 79.0% (95% CI, 69.7-86.5%; PR 70%, PRwL 9%) and a median PFS of 16.8 months (95% CI, 13.2-18.7) was observed in the subgroup of patients who had previously received both a BTK inhibitor and a BCL2 inhibitor (**Table 1**) (93). 37.8% (84/222) of the treated patients exhibit a BTKC481 mutation. Notably, the ORR of patients with a BTKC481 mutation was with 89% (95% CI, 90-95%) significantly higher compared to the ORR of CLL patients without BTKC481 mutation (74%; 95% CI, 64-82%). Moreover, pirtobrutinib is well tolerated in most CLL patients. Only 2.6% discontinued pirtobrutinib therapy due to treatment-related adverse events (93). An ongoing phase III clinical trial is currently comparing the efficacy of pirtobrutinib in combination with venetoclax and rituximab to the standard therapy venetoclax-rituximab in previously treated R/R CLL patients (NCT04965493).

##### 3.5.4.2 Nembtabrutinib

Nemtabrutinib (ARQ-351) is a reversible, non-covalent BTK inhibitor that binds and inhibits the kinase activity of BTK independent of the C481 residue. As a result, nemtabrutinib targets both, normal BTK and the C481-mutated forms of BTK. In preclinical studies, nembtabrutinib increased survival over ibrutinib in Eμ-TCL1 mouse models and was able to suppresses BCR-induced activation of PLCγ_2_ and BTK-C481S mutants, occurring in patients with clinical resistance to ibrutinib (150). In a first-in-human phase I clinical study, nembtabrutinib showed preliminary efficacy in a patient population with advanced R/R CLL. An ORR of 75% (PR) was reached by 8 CLL patients treated at 65mg once daily (n = 8), including 6 patients with a BTK mutation. Median PFS was not reached (95% CI, 16.7 months – NR) (**Table 1**) (94). Another phase I/II study of nemtabrutinib in R/R B-cell malignancies reported an ORR of 56% (95% CI, 42 – 69%; CR 3.5%, PR 26.3% PRwL 26.3%) with a median PFS of 26.3 months (95% CI, 10.1 - NR) (**Table 1**) (95). Notably, 95% of CLL patients received prior treatment with a covalent BTK inhibitor (cBTKi) and 42% had prior cBTKi and BCL2 inhibitor therapy. Moreover, 63% of the CLL study population exhibited a BTK-C481S mutation (95). In general, nembtabrutinib shows promising efficacy in the treatment of high-risk CLL patients with clinical resistance/relapse to previous therapy and successfully targets BTK-C481 mutations facilitating CLL cell resistance to several covalent BTK inhibitors. Moreover, the clinical safety was manageable with 11.4% (94) and 13% (95) of patients, respectively, that discontinued treatment due to treatment-related adverse events. An ongoing, open-label, phase III trial is presently investigating the safety and efficacy of nemtabrutinib in combination with venetoclax as second-line or later therapy for R/R CLL patients (NCT05947851).

##### 3.5.4.3 Vecabrutinib

In a preclinical characterization using an Eμ-TCL1 mouse model, vecabrutinib (SNS-062) significantly reduced tumor burden and improved animal survival (151). However, first results of a phase Ib clinical study (NCT03037645) in CLL patients with or without BTK mutation reveal that vecabrutinib did not translate to such a strong response as expected. In general, vecabrutinib was well-tolerated, but merely resulted in modest clinical benefit, with 0.4% PR (1/30) and 37% (11/30) of the CLL patients having a stable disease (**Table 1**) (154). Despite strong preclinical evidence, the efficacy of vecabrutinib was not sufficient to combat CLL in refractory patients. Consequently, the clinical development and evaluation of vecabrutinib in the treatment of B-cell malignancies was terminated (154).

#### 3.5.5 Emerging resistance to novel BTK inhibitors

Despite the promising results of the novel noncovalent BTK inhibitors, mechanisms of resistance to these drugs could already be observed. Similar to ibrutinib-resistance, mutations in PLCγ_2_ and BTK are the predominant resistance mechanisms to acalabrutinib treatment. BTK C481 mutations occurred in 43% of acalabrutinib-treated CLL patients at the time of disease progression, T474I mutation in 21% and PLCγ_2_ mutations were found 29% (155). On-target BTK mutations (e.g. A428D, V416L, T474I, M437R, L528W) and PLCγ_2_ mutations allow CLL cells to escape the BTK inhibitory effects in CLL patients treated with the noncovalent inhibitor pirtobrutinib (156). Recent data, presented at the ASH meeting 2023, show that in all CLL patients that had a BTK C481S mutation prior to pirtobrutinib therapy, the C481S mutation declines on pirtobrutinib treatment. However, at disease progression this mutation is replaced by a T474I BTK mutation in 3 of 5 patients, while the BTK L528W mutation, that inactivates kinase function is observed in one of 5 patients (157). The kinase-inactive mutation BTK L528W was also enriched in CLL patients, which acquired resistance to the second-generation BTK inhibitor zanubrutinib (158). Similarly, BTK plus PLCγ_2_ mutations, including mutations in the BTK amino acids L528, A428, and V416, were discovered in REC-1 mantle cell lymphoma (MCL) cell lines, which are resistant to vecabrutinib, pirtobrutinib, and fenebrutinib. Interestingly, only REC-1 cells resistant to nemtabrutinib acquired no BTK or PLCγ_2_ mutation, suggesting a different mechanism in the development of resistance (159).

### 3.6 MAPK signaling

The mitogen-activated protein kinase (MAPK) signaling pathway is activated downstream of the BCR and plays a pivotal role in the regulation of cell differentiation, proliferation, survival, plus cell migration (160). Nearly half of CLL patients show activated MAPK signaling, suggesting a pathogenic role of this pathway in CLL (161). In addition, several studies identified activated MAPK signaling as a key oncogenic driver of CLL development and progression, with approximately 5 – 8% of CLL patients harboring at least a single genetic mutation in this pathway. Mutations of the MAPK signaling pathway include the RAS genes (NRAS, KRAS), BRAF, and the novel putative driver MAP2K1 (162, 163). Furthermore, CLL patients carrying such mutations frequently correlate with an aggressive course of disease, exhibiting adverse biological characteristics like an increased CD49d, ZAP-70 expression, trisomy 12 or unmutated IGHV regions and also show a significantly shorter treatment-free survival (163, 164).

Despite its activation in CLL, targeting MAPK signaling does not show significant effects on CLL viability. Paradoxically, MAPK signaling inhibitors promote MAPK signaling activity, reduce the expression of negative modulators of their pathway, and augment AKT-mediated signaling (165). In line with this, genetic deletion or inhibition of the inhibitory phosphatases DUSP1 or DUSP6, which are negatively regulating MAPK signaling, results in reduced CLL cell survival. Apoptosis following DUSP1/6 inhibition was also evident in drug-resistant CLL (166). This appears surprising since MAPK activity is actually well-known for its effects in promoting cell survival. But the B cell-specific physiological effects of the MAPK pathway vary to a great extent. For example, according to the cell type and stimuli, activation of a specific MAPK, named extracellular signal-regulated kinase (ERK), can also trigger apoptotic processes causing cell death (167). Similarly, active ERK1/2 is also associated with cell death during the B cell negative selection, which serves to avert autoimmunity (168). In these cases, acute activation of MAPK signaling promotes the aggregation of mitochondrial reactive oxygen species, thereby inducing cell death mediated by the DNA damage response (167, 168). This phenomenon was also observed after DUSP1/6 inhibition in CLL (166), which supports an important role of DUSP1/6-mediated negative regulation of MAPK signaling in CLL cells survival and proposes DUSP1/6 inhibition along with subsequent over-active MAPK signaling as a potential new CLL therapy approach. In contrast, the MEK1/2 inhibitor binimetinib in monotherapy or combined with the BCL-2 inhibitor venetoclax shows great effectiveness in causing CLL cell death (169). Besides, CLL cells with trisomy 12 are susceptible to ERK and MEK inhibition (170). Furthermore, the ERK inhibitor rulixertinib reduces ERK phosphorylation in MAPK-mutant CLL clones (163). This suggests a dual role of MAPK activity in CLL, which may depend on the CLL subset/cell type.

### 3.7 NF-κB signaling

The nuclear factor κ-light-chain-enhancer of activated B cells (NF-κB) is retained in an inactive state by inhibitor proteins (IκB). Activation of the IκB kinase complex (IKK) leads to IκBs phosphorylation and subsequent degradation, which finally results in NF-κB translocation into the nucleus to induce target gene transcription (**Figure 4**) (171, 172). In B cells, NF-κB mediated transcription can be activated by several upstream signaling pathways including inflammatory cytokines, BCRs, toll-like receptors (TLR) or TNF receptors, like the B cell-activating factor receptor (BAFFR) or CD40 ligand. Activated NF-κB controls multiple processes, such as differentiation, cell cycle progression, and survival (171). Moreover, NF-κB signaling is found to be constitutively activated in CLL patients, indicating that aberrant NF-κB activation plays a crucial role in CLL pathogenesis and progression (13). In CLL cells, activation of the BCR successively increased the NF-κB mediated transcription, indicated by a 23% higher detectable target gene expression (173). Moreover, a few recurrently mutated genes involved in the activation of NF-κB were observed in CLL at a low frequency (> 5%) (i.e. mutations in MYD88, BIRC3, NFKBIE) (174–177). The mutated myeloid differentiation primary response 88 (MYD88) protein imitates constitutively active TLR signaling and thus intensifies BCR–mediated NF-κB signaling (177). The loss-of-function mutation in the gene coding for the baculoviral IAP repeat containing 3 (BIRC3) prevents negative regulation of the MAP3K14/NF-κB inducing kinase (NIK), a key activator of NF-κB signaling (174). The mutation in the inhibitory IκBϵ molecule encoding gene NFKBIE is associated with significantly enhanced NF-κB activation and is most frequently found in poor-prognostic subgroups of CLL (176). It was shown that cross-talk of CLL cells with the tumor microenvironment results in NF-κB activation, which provides pro-survival signals to the malignant CLL clones by increasing the expression of various anti-apoptotic genes (13, 178). Thus, NF-κB activity correlates with a dismal CLL outcome and represents an essential mechanism of CLL resistance (13, 178). Most interestingly, resistance mutations to BTK inhibitors were revealed to arise only through NF-κB and not via PI3K-RAS-MAPK-mediated signaling of the BCR pathway. Following BTK inhibition, CLL cells only select gain-of-function alterations that are mediated by NF-κB signaling, highlighting BTK’s significant role in BCR-induced activation of NF-κB (179). Hence, targeting NF-κB signaling represents a potential future therapeutic approach to overcome CLL resistance which is supported by NF-κB survival signaling originating from the protective tumor microenvironment. A first *in vitro* study shows promising effects in targeting the NF-κB inducing kinase (NIK) by the inhibitor CW15337 in CLL cell clones (180).

## 4 Tumor microenvironment and BCR-associated pathways in CLL

In addition to the signals originating from the BCR, CLL cell survival depends on various co-stimulatory signals, which can occur through direct cellular interactions or via soluble factors. These signals are essential for CLL cell survival by facilitating proliferation as well as migration and homing of the malignant cells to protective niches where they are able to undergo cell division. One BCR-associated pathway with continuously rising importance in regulating migration and homing of CLL is the CXCR4 signaling pathway.

### 4.1 CXCR4 signaling

The C-X-C motif chemokine receptor 4 (CXCR4) regulates the movement of B cells toward the chemokine C-X-C motif ligand 12 (CXCL12), its corresponding chemokine ligand. Interaction of CXCL12 with CXCR4 triggers the activation of several downstream pathways such as MAPK/ERK, PI3K/AKT, PLCγ/Ca^2+^ and NF-κB signaling (**Figure 4**) (181, 182). For CLL cells, CXCR4 expression is critical for the migration toward specific niches, where the leukemic cells are protected by a survival- and growth-promoting microenvironment (183). This is why in CLL patients, CXCR4 is upregulated and associated with adverse prognosis. In line with this, CLL patients exhibiting low CXCR4 levels are associated with good prognosis as well as a significantly decreased risk of disease progression (184). An oncogenic hyperactivated form of CXCR4 in Eµ-TCL1 mice was described to collaborate with TCL1 in accelerating the progression of CLL (182). Upon ibrutinib treatment, a downmodulation of CXCR4 expression levels and CXCR4 signal inhibition in CLL cells could be identified in Eµ-TCL1 mice (185). Lately, it was reported that CRISPR-Cas9 induced disruption of CXCR4 signaling significantly affects not only the migration and homing of CLL cells with RT, but also reduces cell growth in murine and patient-derived xenograft models and impairs BCR-mediated signaling (186). This is a first evidence that targeting the CXCR4 pathway possibly represent a potent new therapeutic target in CLL patients with or without RT. Regarding the emerging importance of CXCR4 in CLL progression and survival, CXCR4 inhibitors are presently investigated in clinical studies (187).

In addition, it was revealed that according to CXCR4 expression in combination with CD5 (a surface molecule characteristically expressed on CLL B cells), CLL clones can be defined into subgroups varying in the time elapsed since the last cell division (also called “age”): the newly originated, proliferative fraction (PF; CXCR4^Dim^CD5^Bright^), the double dim fraction (DDF; CXCR4^Dim^CD5^Dim^), the intermediate fraction (IF; CXCR4^Int^CD5^Int^), the double bright fraction (DBF; CXCR4^Bright^CD5^Bright^) and the resting fraction (RF; CXCR4^Bright^CD5^Dim^) (188–190). The last-born cells are thought to enter the circulation as PFs and from there transition to either a low CD5 (DDF) or a high CXCR4 (IF and DBF) phenotype, eventually converging into RFs (190). Besides, the fractions also differ in smIgM and smIgD BCR densities, since young cells of the PF show high IgM/IgD surface expression, whereas cells with low IgM/IgD expression were the oldest (RF) (190). While in CLL patients the peripheral blood mainly consists of quiescent CLL cells of the RF, the CLL cells residing in LNs are actively proliferating cells of the PF (CXCR4^Dim^CD5^Bright^) (189, 191). In compliance, gene expression analyses revealed that LNs are specific sites, where the upregulation of genes associated with BCR activation as well as CLL cell proliferation takes place (13). Furthermore, the high proliferation rate in LNs correlates with aggressive disease, rapid lymphocyte doubling, and shorter treatment-free survival compared to CLL with low growth rates (13, 191). Most interestingly, the different intraclonal fractions show variable susceptibility to CLL therapy, since older CXCR4-positive CLL cells (RF and DBF) were observed to be less susceptible to *in vivo* inhibition by ibrutinib relative to younger cells (190). This points out the necessity to develop new treatment strategies specifically targeting all points in the life cycle of the various intraclonal fractions of a CLL clone.

## 5 BCR signaling in the CLL cell metabolism

Deregulated cellular energy metabolism is a well-known hallmark of cancer. Like all malignant cells, CLL cells make adaptations to meet their increased metabolic needs. The mechanistic target of rapamycin (mTOR) complex is crucial for the coordination of energy, oxygen, nutrient and growth factor availability in the cell as well as the regulation of cellular growth and survival (192). mTOR forms two structurally and functionally unique complexes, mTORC1 and mTORC2. Although both complexes are crucial mediators of cellular metabolism, solely mTORC1 is directly activated by nutrient, oxygen, and energy availability, which is ultimately resulting in DNA, protein, and lipid synthesis as well as cellular growth (193). By inducing the activation of mTORC1, BCR signaling is (among others) directly implicated in the regulation of B cell metabolism. The tuberous sclerosis complex 1/2 (TSC1/2) negatively regulates mTORC1 by inhibiting RHEB GTPase activity, which is required to induce mTORC1 activation. BCR-mediated activation of the MAPK and PI3K/AKT signaling cascades leads to the phosphorylation and inhibition of TSC2, which ultimately results in the activation of mTORC1 (194).

In CLL, BCR signaling was found to regulate cellular metabolism via the PI3K/AKT/mTOR signaling axis. Genetic deletion as well as inhibition of PI3Kδ results in a significant reduction of the metabolic flux in CLL cells (195). Moreover, metabolic flux analysis of 140 CLL patients revealed that patients that are diseased with the more aggressive form of U-CLL exhibit significantly higher glycolytic activity compared to M-CLL patients. These results indicate that the IGHV mutational status of CLL cells is directly linked to their glycolytic activity, most likely by BCR-mediated signaling (196). Similar to the IGHV mutational status, glycolytic capacity was found to be a reliable predictor of overall survival in CLL patients (196). Moreover, the proliferative drive in CLL cells is associated with high MYC and mTOR activity promoting mitochondrial biogenesis and leading to increased oxidative phosphorylation (OXPHOS). Increased MYC-mTOR-OXPHOS activity cooperates to drive cell growth and meet the increased energy needs in CLL (197). Due to the increased PI3K/AKT/mTOR pathway activity in CLL, studies targeting PI3K/AKT/mTOR signaling indicated pro-apoptotic effects in the treatment of CLL and other B cell leukemias (198). Thus, PI3K/AKT/mTOR signaling may present a future therapeutic target in the treatment of CLL, probably also in combination with already existing therapeutics. For example, combined mTOR and electron transport chain inhibition were found to synergistically counteract venetoclax resistance in CLL (199). This synergistic effect may provide an opportunity to enhance the efficacy of the BCL2 inhibitor venetoclax, which is frequently used in the treatment of CLL.

## 6 Conclusion

Our understanding of the CLL pathogenesis regarding BCR-mediated signaling, tumor microenvironment, and co-stimulatory signals has markedly improved during the last years. Furthermore, the range of therapeutic options to treat CLL has considerably increased. The development of next-generation drugs targeting BCR signaling crucial for CLL cell pathogenesis and survival has significantly ameliorated the clinical course of CLL patients. Several inhibitors targeting BCR downstream signaling pathways are already in clinical use and show high efficacy in CLL therapy (16–18, 74). Among all therapeutics targeting BCR-signaling, BTK-targeting inhibitors show the most beneficial clinical responses in the treatment of CLL. With an ORR of 95.4% (95% CI, 92.1-97.6%), a CR of 71.5% (95% CI, 65.6%, 76.9%) and a median 3-year PFS rate of 97.2% (HR = 0.13; 95% CI, 0.07-0.24), the combination of the BTK inhibitor ibrutinib with the BCL2 inhibitor venetoclax indicates superior clinical efficacy and suggests a strong synergy of BCL2 and BCR-dependent pathways (83). In most cases, disease progression is a result of specifically acquired mutations that allow the CLL cell to escape the inhibitory mechanism of the therapeutic agent. Due to constant research and improvement of the current therapeutics, novel drug combinations as well as next-generation inhibitors are also able to partially overcome therapy resistance in refractory CLL. Reversible, non-covalent BTK-inhibitors, such as pirtobrutinib or nemtabrutinib for instance, successfully target CLL cells exhibiting a BTK-C481 mutation and show promising efficacy in the treatment of CLL patients that have developed clinical resistance to ibrutinib therapy (93, 94). However, despite the immense progress in CLL research, no agent has clearly demonstrated efficacy in R/R CLL patients in the long run. The emerging importance of the tumor-supporting microenvironment in CLL progression and survival represents a novel point of action in the treatment of CLL. CXCR4-targeting inhibitors may present a promising mechanism to enhance the efficacy of existing treatment options by preventing CLL cell migration to these protective niches. Moreover, the variable susceptibility to CLL therapy of the different intraclonal fractions (187) points out the necessity to develop new treatment strategies specifically targeting all points in the life cycle of a CLL clone. Thus, continuous research in the field of CLL to understand the molecular mechanisms of leukemic transformation as well as CLL cell survival is of utmost importance to identify new drug targets or combinations and mechanisms of drug action in the future.

## Author contributions

VS: Writing – review & editing. EH: Supervision, Writing – review & editing.
